# *Anoplophorahuangjianbini* sp. n. from Fujian and Guangxi, China (Coleoptera: Cerambycidae: Lamiinae)

**DOI:** 10.3897/BDJ.9.e70936

**Published:** 2021-08-12

**Authors:** Cheng-Bin Wang, Li He

**Affiliations:** 1 Engineering Research Center for Forest and Grassland Disaster Prevention and Reduction, Mianyang Normal University, 166 Mianxing West Road, Mianyang, China Engineering Research Center for Forest and Grassland Disaster Prevention and Reduction, Mianyang Normal University, 166 Mianxing West Road Mianyang China; 2 No.66, 5th Shuangcheng Road, Chenghua District, Chengdu, China No.66, 5th Shuangcheng Road, Chenghua District Chengdu China

**Keywords:** longhorn beetle, Lamiini, taxonomy, new species, Oriental Region

## Abstract

**Background:**

The genus *Anoplophora* Hope, 1839 (Coleoptera: Cerambycidae: Lamiinae: Lamiini) includes 47 species (without subspecies) occurring in East, South and Southeast Asia. Amongst them, 38 species are known from CHINA. Members of this genus are familiar to Chinese people with a widely-used common name: “星天牛 [starry longhorn beetle]”. *Anoplophora* species have great economic importance, attacking and damaging numerous hardwood trees and some coniferous trees.

**New information:**

A new species of starry longhorn beetle, *Anoplophorahuangjianbini* **sp. n.** (Coleoptera: Cerambycidae: Lamiinae: Lamiini) is described from Fujian and Guangxi, CHINA. Diagnostic characters of the new species are illustrated and comparison with closely-related congeners is provided.

## Introduction

The genus *Anoplophora* Hope, 1839 (Coleoptera: Cerambycidae: Lamiinae: Lamiini) can be separated from allied genera by a combination of the following characters: female sternite VII with lateral notches approximately at the level where the ventral apodeme of sternite VIII attaches; mesotergum consisting of two overlapping plates (as in *Monochamus* and *Eupromus*), but overlap evenly and broadly convex laterally, with small notches extending laterally anterior to the base of scutellum; antennal scape with small to large apical cicatrix; and pronotum with posteromedial callus in most species (Lingafelter and Hoebeke 2002). Since the monograph of “Revision of the genus *Anoplophora* (Coleoptera: Cerambycidae)” by Lingafelter and Hoebeke (2002), ten species and two subspecies have been included in *Anoplophora*. *Anoplophorasiderea* Bi, Chen & Ohbayashi, 2020, *A.fanjingensis* Yang, Yang & Tian, 2020 and *A.puxian* Wang & He, 2021 are the latest three species contributing to the genus (Bi et al. 2020, Yang et al. 2020, Wang and He 2021). Shortly after our description of *Anoplophorapuxian*, Mr. Jian-Bin Huang (Nanping, CHINA) presented us with a pair of specimens from Fujian, CHINA, which were identified as an unkown species of *Anoplophora*. Later, more specimens of this species were available to us from different sources, including those from Guangxi, CHINA. Herein, we describe and illustrate it under the name of *Anoplophorahuangjianbini* **sp. n.** Therefore, the number of the *Anoplophora* species from CHINA comes to 39 (without subspecies) (Lin and Tavakilian 2019, Lin 2020, Wang and He 2021). Important morphological characters of the new species are illustrated and its differential diagnosis from related species is provided.

## Materials and methods

Specimens were relaxed and softened in a HH-2 digital homoeothermic water bath at 44.4℃ for 14 hours, then transferred to distilled water to clean, observe and dissect. In order to examine the genitalia, the abdomen was detached and treated with a 10% solution of potassium hydroxide (KOH) for 12 hours, then transferred to distilled water to remove the remaining KOH and stop any further bleaching. After examination, the body parts were mounted on a glass slide with Euparal Mounting Medium for future studies. Habitus images were taken using a Canon 50D DSLR with a Canon EF 100 mm f/2.8L IS USM lens and a Canon MT-24EX Macro Twin Lite Flash was used as the light source. Images of the morphological details were taken using a Canon macro photo lens MP-E 65 mm on a Canon 5DsR. Images of the same specimen/structure at different focal planes were combined using Zerene Stacker 1.04 stacking software. Adobe Photoshop CS6 was used for post-processing. The terminology adopted in this paper for external features of the body and genitalia follows Lawrence et al. 2011.

The material examined for this study is deposited in the following institutional and private collections: **CCZC**: collection of Chao Zhou, Chengdu, CHINA; **CLGS**: Collection of Liang Guo, Sanming, CHINA; **CLHC**: Collection of Li He, Chengdu, CHINA; **CJBH**: Collection of Jian-Bin Huang, Nanping, CHINA; **CPYL**: Collection of Peng-Yu Liu, Nanping, CHINA; **CTLH**: Collection of Tian-Long He, Huainan, CHINA; **MYNU**: insect collection of Mianyang Normal University, Mianyang, CHINA.

The following material was studied for comparison: ***Anoplophorachiangi* Hua & Zhang, 1991. CHINA**: 1♂(Fig. 2D), Guizhou, Tongren City, Jiangkou County, Mount Fanjing, Huixiangping [回香坪], alt. 2100 m, 25.VII.2016, Zi-Hao Yang leg. (CLHC). ***Anoplophoraelegans* (Gahan, 1888). CHINA**: 1♂1♀, Yunnan, Honghe Hani & Yi Autonomous Prefecture, Lvchun County, Huanglianshan National Nature Reserve [黄连山国家级自然保护区], alt. 1850 m, VI.2019, Tian-Long He leg. (CTLH); 1♀, Yunnan, Xishuangbanna Dai Autonomous Prefecture, Mengla County [勐腊县], alt. 1200 m, VIII.2020, Zhong-Xiong Fu leg. (CLHC); 1♀, ditto except alt. 800 m, Yi Li leg. (CLHC); 1♂, Yunnan, Baoshan City, Tengchong, Yunfeng mountain [云峰山], alt. 971 m, 15.VII.2021, Chen-Yan Jin leg. (CLHC); 3♀♀, Guangxi, Fangchenggang City, Shangsi County, Shiwandashan National Forest Park [十万大山国家森林公园], VI.2021, local people leg. (CLHC); **LAOS**: 1♂(Fig. 2B), Mt. Phu Pham, alt. 2060 m, 15.V.2019, local people leg. (CLHC); **VIETNAM**: 1♂, Yenbai, Mucang Chai, V.2019, local people leg. (CLHC). ***Anoplophoraimitator*** **(White, 1858). CHINA**: 1♂(Fig. 2A)1♀, Fujian, Fuzhou City, Lianjiang County, Danyang Town [丹阳镇], 25.VII.2020, Fu-Xing Chen leg. (CLHC); 1♂1♀, Fujian, Ningde City, Jiaocheng District [蕉城区], alt. 60 m, 20.VII.2019, Ben-Fu Miao leg. (CLHC). ***Anoplophorasimilis* (Gahan, 1900). CHINA**: 1♂(Fig. 2C), Hainan, Ledong Li Autonomous County, Jianfeng Town, Jianfengling National Forest Park, Mingfenggu [鸣凤谷], 8.74192°N, 108.84834°E, alt. 945 m, 24.V.2014, Bin Liu leg. (CLHC).

Measurement criteria in millimetres (mm) are as follows: **antennal length**: length between the base and the apex of antenna; **body length**: length between the head vertex and the elytral apex along the mid-line; **elytral length**: length between the basal border and the apex of elytra along suture; **head length**: length between the anterior apex of clypeus and the posterior margin of occiput along the midline; **head width**: widest part of head (including eyes); **humeral width**: width across elytral humeri; **pronotal length**: length of the pronotum along the mid-line; **pronotal apical width**: width across the apical margin of pronotum; **pronotal basal width**: width across the basal margin of pronotum; **pronotal maximum width**: widest part of pronotum (including lateral spines).

## Taxon treatments

### Anoplophora huangjianbini

Wang & He, 2021 sp. n.

C8622FE1-9F27-5969-9896-860DA1303665

5DF3AD6C-7A89-487E-9684-DFF86E318C61

#### Materials

- **Type status:**Holotype. **Occurrence:** recordedBy: Jian-Bin Huang; individualCount: 1; sex: male; **Location:** country: CHINA; stateProvince: Fujian; verbatimLocality: Sanming City, Sha County, Luoboding [三明市沙县锣钹顶]; verbatimElevation: 1360 m; verbatimLatitude: N26.25843°; verbatimLongitude: E117.73736°; **Event:** year: 2018; month: 7; **Record Level:** institutionCode: MYNU
- **Type status:**Paratype. **Occurrence:** recordedBy: Jian-Bin Huang; individualCount: 1; sex: female; **Location:** country: CHINA; stateProvince: Fujian; verbatimLocality: Sanming City, Sha County, Luoboding [三明市沙县锣钹顶]; verbatimElevation: 1360 m; verbatimLatitude: N26.25843°; verbatimLongitude: E117.73736°; **Event:** year: 2018; month: 7; **Record Level:** institutionCode: MYNU
- **Type status:**Paratype. **Occurrence:** recordedBy: Yong Li; individualCount: 1; sex: male; **Location:** country: CHINA; stateProvince: Fujian; verbatimLocality: Sanming City, Sha County, Luoboding [三明市沙县锣钹顶]; verbatimElevation: 1360 m; verbatimLatitude: N26.25843°; verbatimLongitude: E117.73736°; **Event:** year: 2018; month: 7; **Record Level:** collectionCode: CJBH
- **Type status:**Paratype. **Occurrence:** recordedBy: Liang Guo; individualCount: 1; sex: female; **Location:** country: CHINA; stateProvince: Fujian; verbatimLocality: Sanming City, Tianbaoyan Nature Reserve [三明市天宝岩自然保护区]; verbatimElevation: 1100 m; **Event:** year: 2015; month: 6; day: 19; **Record Level:** collectionCode: CLGS
- **Type status:**Paratype. **Occurrence:** recordedBy: Jian-Bin Huang; individualCount: 1; sex: male; **Location:** country: CHINA; stateProvince: Fujian; verbatimLocality: Ningde City, Gutian County, Shitashan [宁德市古田县石塔山]; verbatimElevation: 1340 m; verbatimLatitude: N26.84247°; verbatimLongitude: E118.63524°; **Event:** year: 2020; month: 7; day: 9; **Record Level:** collectionCode: CJBH
- **Type status:**Paratype. **Occurrence:** recordedBy: Peng-Yu Liu; individualCount: 1; sex: male; **Location:** country: CHINA; stateProvince: Fujian; verbatimLocality: Ningde City, Gutian County [宁德市古田县]; verbatimElevation: 1550 m; **Event:** year: 2021; month: 7; day: 18; **Record Level:** collectionCode: CPYL
- **Type status:**Paratype. **Occurrence:** recordedBy: Jian-Bin Huang; individualCount: 1; sex: female; **Location:** country: CHINA; stateProvince: Fujian; verbatimLocality: Quanzhou City, Daiyun Mountain, hiking trail [泉州市戴云山登山步道]; verbatimElevation: 1520 m; **Event:** year: 2021; month: 7; day: 4; **Record Level:** collectionCode: CLHC
- **Type status:**Paratype. **Occurrence:** recordedBy: local people; individualCount: 1; sex: male; **Location:** country: CHINA; stateProvince: Guangxi; verbatimLocality: Nanning City, Wuming County, Damingshan [南宁市武鸣县大明山]; verbatimElevation: 1600 m; **Event:** year: 2005; month: 7; **Record Level:** collectionCode: CJBH
- **Type status:**Paratype. **Occurrence:** recordedBy: local people; individualCount: 1; sex: male; **Location:** country: CHINA; stateProvince: Guangxi; verbatimLocality: Laibin City, Jinxiu County, Changtong Township, Daojiang Village, Pingbantun [来宾市金秀县长垌乡道江村平办屯]; verbatimElevation: 1375 m; verbatimLatitude: N24.09509°; verbatimLongitude: E110.18344°; **Event:** year: 2015; month: 7; day: 8; **Record Level:** collectionID: CLHC
- **Type status:**Paratype. **Occurrence:** recordedBy: Chun-Fu Feng; individualCount: 1; sex: female; **Location:** country: CHINA; stateProvince: Guangxi; verbatimLocality: Laibin City, Jinxiu County, Changtong Township [来宾市金秀县长垌乡]; **Event:** year: 2020; month: 5; **Record Level:** collectionCode: CCZC
- **Type status:**Paratype. **Occurrence:** recordedBy: local people; individualCount: 1; sex: female; **Location:** country: CHINA; stateProvince: Guangxi; verbatimLocality: Laibin City, Jinxiu County, Shengtangshan [来宾市金秀县圣堂山]; verbatimElevation: 1500 m; **Event:** year: 2018; month: 7; **Record Level:** collectionCode: CJBH
- **Type status:**Paratype. **Occurrence:** recordedBy: Huang-Shun Xi; individualCount: 1; sex: male; **Location:** country: CHINA; stateProvince: Guangxi; verbatimLocality: Laibin City, Jinxiu County, Dayaoshan Mountain [来宾市金秀县大瑶山]; verbatimElevation: 1350 m; **Event:** year: 2018; month: 7; day: 2; **Record Level:** collectionCode: CTLH

#### Description

**Holotype male**. Body 28.6 mm long, widest just after elytral humeri (10.8 mm). Length of different body parts (mm): head (3.3), antenna (54.5), pronotum (5.1), elytra (20.4); width: head (5.9), pronotal apex (6.2), pronotal base (6.6), elytral humeri (10.1).

**Figure 1.**
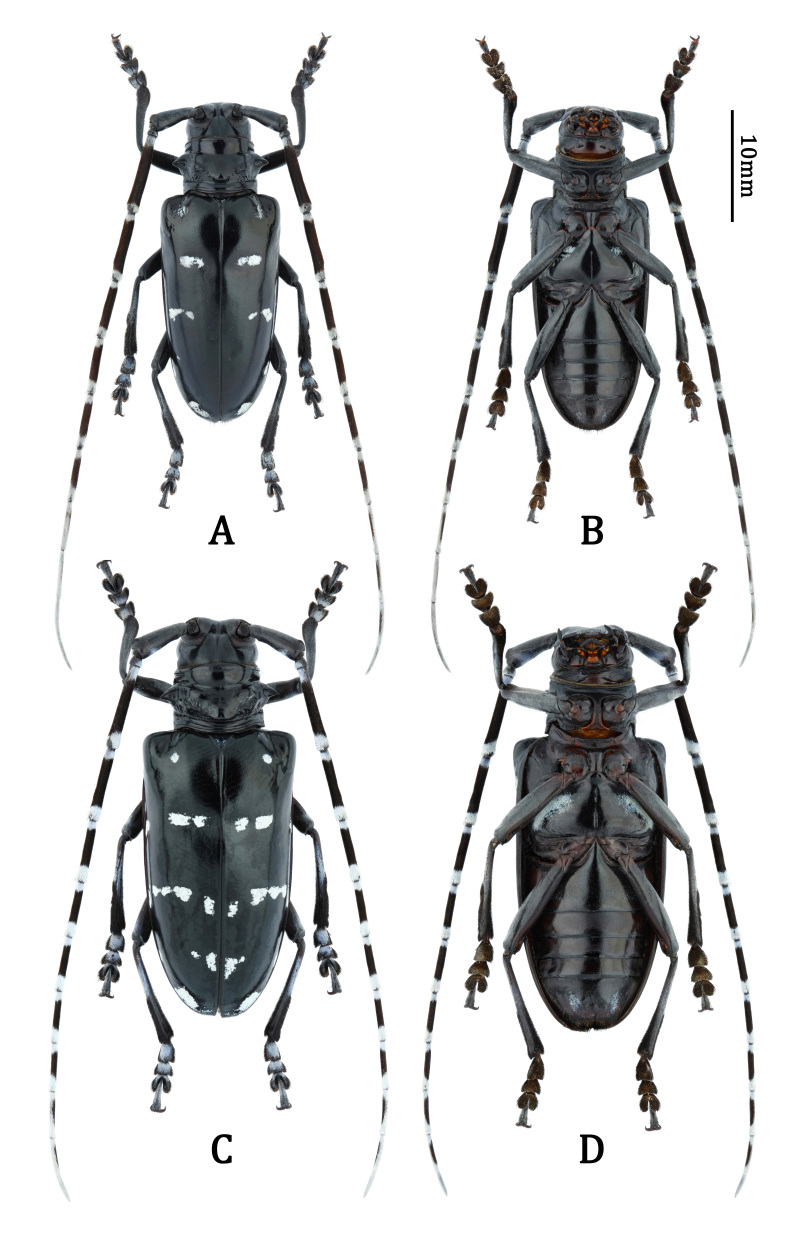
Habitus of *Anoplophorahuangjianbini* **sp. n.**: **A, B.** male, holotype; **C, D.** female, paratype. **A, C.** dorsal view; **B, D.** ventral view.

**Habitus** (Fig. 1A and B). Body oval. Integumentary colour of body and appendages blackish; eyes black; elytra blackish with weak green sheen. Frons, genae and vertex almost glabrous. Temples moderately clothed with slender, recumbent, pale pubescence. Scape and pedicel densely clothed with short, recumbent, pale pubescence; in addition, pedicel with whitish pubescence at mesial side. Antennomeres III–XI annulated by whitish pubescence at bases and apices and clothed with dark brown pubescence in middle parts; annulations broadening apically and apical three antennomeres almost entirely covered with whitish pubescence, except in middle of mesial side. Pronotum almost glabrous, inserted with several moderately long, suberect, dark brown setae after lateral spines. Scutellum clothed with whitish pubescence. Elytra mostly glabrous; each elytron provided with several small maculae of white pubescence, roughly arranged in four transverse rows and one relatively large macula along apical margin. Ventral surface predominantly clothed with fine pale pubescence, laterals of metasternum and abdominal sternites III–VII provided with maculae of whitish pubescence. Legs mostly clothed with dark brown pubescence, but with bluish-white pubescence on basal half of tibiae and tarsal dorsum.

**Head** subcylindrical, 1.7 times wider than long, widest at posterior margin of lower eye lobes, slightly narrowed posteriorly. Vertex, frons and genae sparsely covered with fine punctures, interstices microreticulate. Anteclypeus membranous, transverse, without setae or evident punctures; anterior margin straight. Frons with fine median groove extending from anterior margin to occiput. Vertex moderately concave; antennal tubercles prominent.

**Mouthparts**. Labrum wider than long, covered with short brownish setae on dorsal surface, especially dense along anterior margin and with long, strong, blackish setae in apicolateral areas; anterior margin gently emarginate. Mandible short, regularly arcuate at outer edge. Maxillary and labial palpi with ultimate palpomeres fusiform, truncated at apices.

**Antennae** moderately long, 1.9 times as long as body, with apical five antennomeres exceeding elytral apices. Antennomeres with length ratio from base to tip: 6.71 – 1.00 – 10.22 – 9.07 – 7.69 – 7.35 – 7.28 – 6.92 – 6.37 – 6.14 – 9.72. Scape subcylindrical, robust, gradually thickening towards apex, with distinct cicatrix. Pedicel knob-like, short, distinctly thinner than scape. Antennomere III the longest, 1.7 times longer than pronotum, 1.5 times longer than scape and 1.1 times longer than IV. Antennomeres III–X straight, gradually decreasing in length. Antennomere XI arcuate, 1.6 times longer than X.

**Pronotum** subcylindrical, slightly widening basally, 0.8 times as long as basal width, widest at lateral spines. Lateral spine conical, with subacute apex laterally directed and slightly retrousse. Posteromedial callus moderately developed and elevated. Surface with a few umbilicated granules and wrinkles between lateral spine and posteromedial callus.

**Scutellum** subtriangular, narrowly rounded at apex. Surface densely and finely punctuated.

**Elytra** semi-oval, 2.0 times as long as humeral width, widest just after humeri. Humeral width 1.5 times wider than pronotal base. Lateral margin gradually narrowing towards conjointly rounded apices; sutural angle round. Surface smooth, without any granules, sparsely covered with fine punctures, interstices microreticulate.

**Legs**. Femora moderately stout; metafemora reaching posterior margin of abdominal sternite VI. Tibiae moderately long; pro- and mesotibiae gently incurvate around apical 1/3; metatibiae straight. Tarsomere I the longest, but not longer than II+III; III distinctly bilobed.

**Ventral side**. Prosternum smooth; prosternal process almost smooth, apically truncated. Mesosternal process with one strong middle tubercle. Metaventrite with fine median groove extending from sub-base to apical 3/7. Metanepisternum wedge-like; anterior margin widely rounded; ventral margin gently incurved at anterior area and slightly wide at posterior area.

**Abdomen**. Abdominal tergite VII (Fig. 3C) subhexagonal, almost simply rounded at posterior margin; tergite VIII (Fig. 4A) subhexagonal, distinctly emarginate at posterior margin, hardly depressed on dorsal surface (Fig. 4C). Abdominal sternites densely and finely punctate; sternite VII (Fig. 3D) subtrapezoidal, slightly emarginate at middle of posterior margin; sternite VIII (Fig. 4B) with sclerotised area rather narrow, widely emarginate at middle of posterior margin. Spiculum gastrale (Fig. 4D and E) with stem 2.1 times longer than branches; stem straight, except hook-like base in lateral view (Fig. 4F).

*Male genitalia*. Tegmen (Fig. 5A and B) widest at basal 3/7, moderately curved ventrally in lateral view (Fig. 5C); parameres 1/4 length of tegmen, moderately elongate, gradually convergent towards round apex, apex with long setae (Fig. 5D). Median lobe (Fig. 5E and F) longer than tegmen, moderately curved ventrally in lateral view (Fig. 5G); dorsal plate (Fig. 5H) abruptly narrowed from subapex, rounded at apical margin; ventral plate (Fig. 5H) distinctly longer than dorsal plate, narrowed from subapex, rounded at apical margin; basal struts (Fig. 5E and F) half length of median lobe.

**Male paratypes.** Body 28.1–28.8 mm long. Five male types have the same body colour, without evident variations. Whitish or white pubescence stained with bluish tint in different degrees. Some males sparsely clothed with white pubescence on anterolateral and lateral areas of pronotum (lost in holotype). Due to the condition of different specimens, whitish or white pubescence or maculae may be distinct, vague or absent.

**Female paratypes.** Body 34.8–35.6 mm long, widest just after elytral humeri (13.8 mm). Length of different body parts (n = 2, average value, mm): head (3.9), antenna (56.1), pronotum (6.2), elytra (25.3); width: head (7.3), pronotal apex (7.6), pronotal base (8.2), elytral humeri (13.3). Antennomeres with length ratio from base to tip: 5.24 – 1.00 – 6.78 – 5.88 – 5.02 – 4.39 – 4.35 – 4.11 – 3.84 – 3.66 – 5.24.

Similar to male in general appearance, but distinct by the following characters: body much larger (Fig. 1C and D); antennae much shorter, about 1.6 times as long as body, with apical four antennomeres exceeding elytral apices; ventral surface with whitish maculae broader; abdominal tergite VII (Fig. 3A) and sternite VII (Fig. 3B) deeply emarginate in middle of posterior margins; spermatheca (Fig. 6A) short, stout, bisinuate, also bisinuate in lateral view (Fig. 6B).

#### Diagnosis

**Figure 2.**
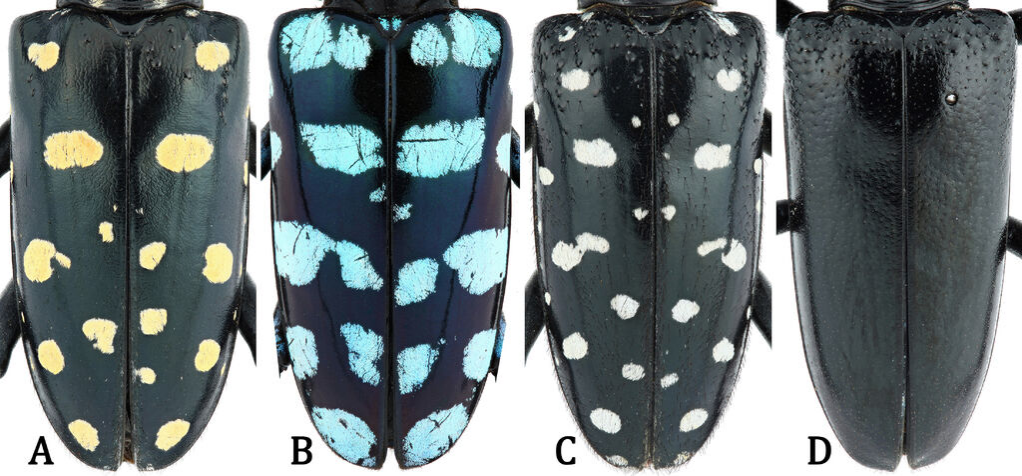
Elytra of *Anoplophora* males, dorsal view: **A.** *A.imitator* (White, 1858) from Fujian, CHINA; **B.** *A.elegans* (Gahan, 1888) from LAOS; **C.** *A.similis* (Gahan, 1900) from Hainan, CHINA; **D.** *A.chiangi* Hua & Zhang, 1991 from Guizhou, CHINA.

**Figure 3.**
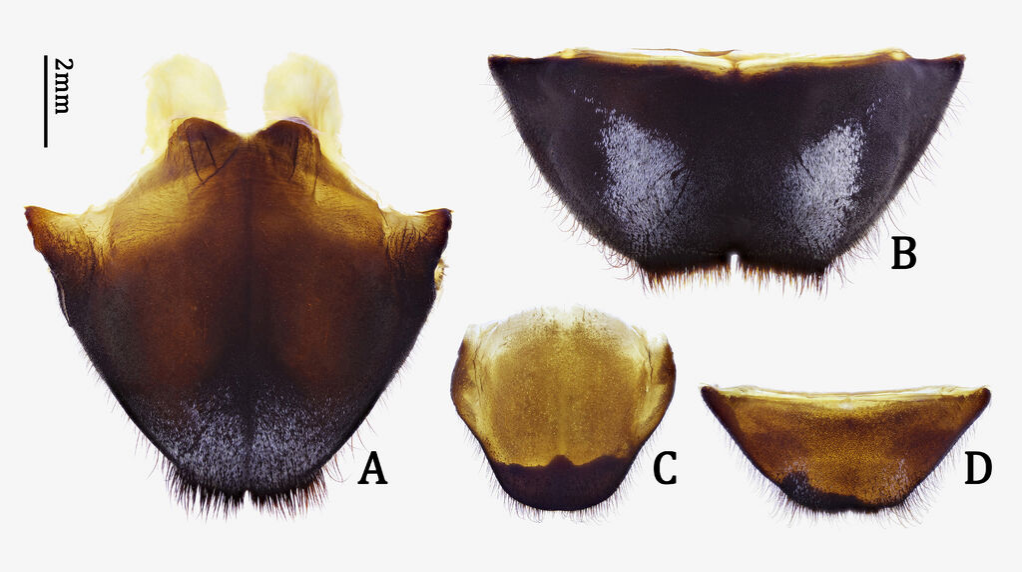
*Anoplophorahuangjianbini***sp. n.**: abdominal tergites VII (**A, C**) and sternites VII (**B, D**) of female, paratype (**A, B**) and male, holotype (**C, D**). **A, C.** dorsal view; **B, D.** ventral view.

**Figure 4.**
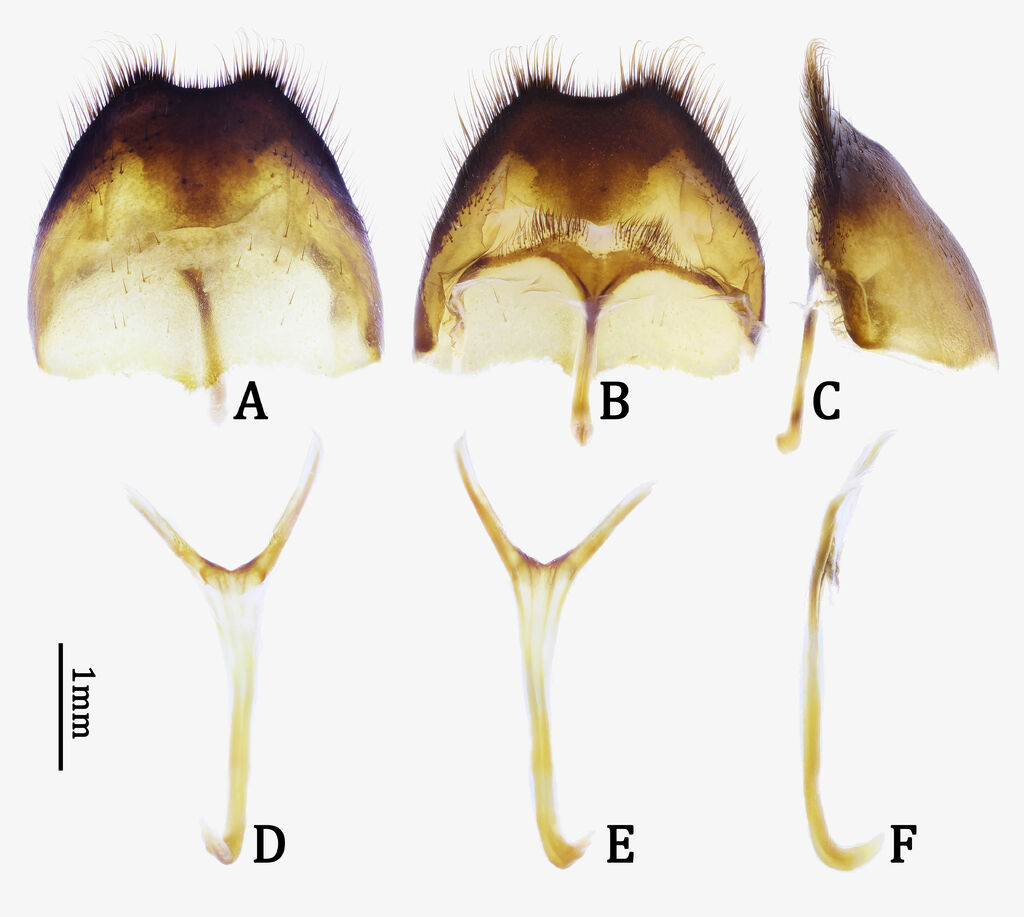
*Anoplophorahuangjianbini***sp. n.**, holotype, male: **A–C.** abdominal segment VIII; **D–F.** spiculum gastrale. **A, D.** dorsal view; **B, E.** ventral view; **C, F.** lateral view.

**Figure 5.**
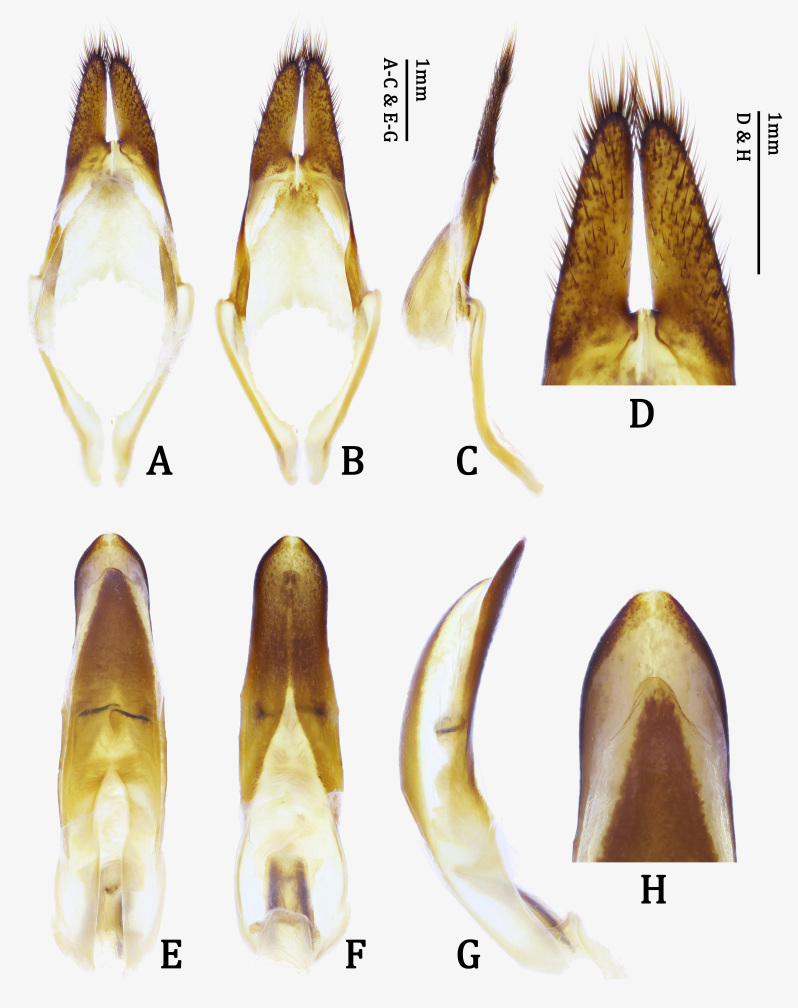
Male genitalia of *Anoplophorahuangjianbini* **sp. n.**, holotype: **A–C.** tegmen; **D.** parameres; **E–G.** median lobe; **H.** apical part of median lobe. **A, E.** dorsal view; **B, F.** ventral view; **C, G.** lateral view; **D, H.** apicodorsal view.

**Figure 6.**
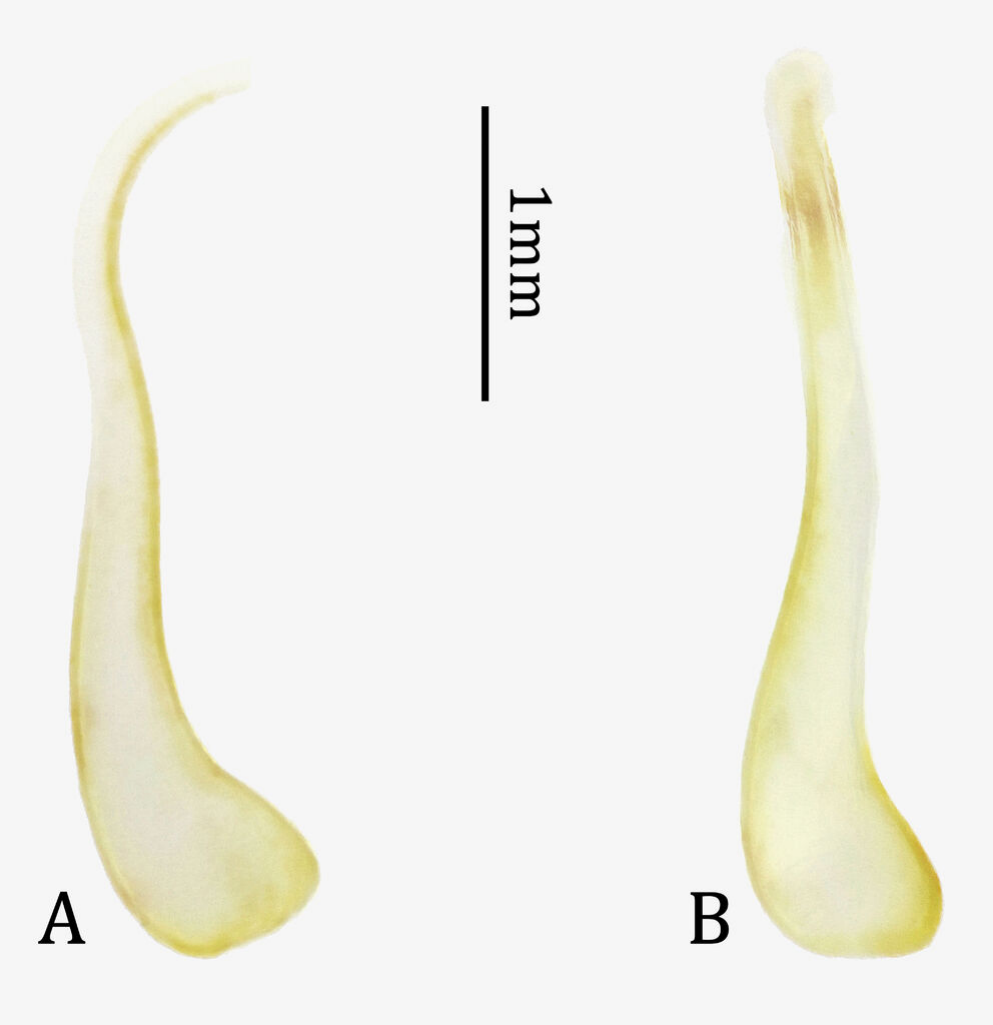
Spermatheca of *Anoplophorahuangjianbini* **sp. n.**, paratype. **A.** dorsal view; **B.** lateral view.

*Anoplophorahuangjianbini***sp. n.** is similar to *A.imitator* (White, 1858) (Fig. 2A), *A.elegans* (Gahan, 1888) (Fig. 2B), *A.similis* (Gahan, 1900) (Fig. 2C), *A.chiangi* Hua & Zhang, 1991 (Fig. 2D) and *A.siderea* Bi, Chen & Ohbayashi, 2020 (figs. 1–3 in Bi et al. 2020) by the distinctive antennal banding, which are annulated with whitish pubescence both at the bases and apices of antennomeres III–XI. However, the new species can be easily separated from them (except *A.elegans*) by the granules (or granulation) lacking on the anterior part of elytra (Fig. 1A and C). For *A.elegans*, it has a totally different pronotal and elytral design, with maculae (or rather bands) much larger/longer and more dense on pronotum, elytra, metasternum and abdominal sternites III–VII; besides, it also has large maculae around gena and frons and on mesepisternum (absent in *A.huangjianbini* **sp. n.**). Moreover, the new species is distinguished from its congeneric species by a combination of the following characters: abdominal tergite VII almost simply rounded at posterior margin (Fig. 3C); tergite VIII distinctly emarginate at posterior margin (Fig. 4A); sternite VIII with sclerotised area rather narrow, widely emarginate at middle of posterior margin (Fig. 4B); spiculum gastrale with stem straight, except hook-like base in lateral view (Fig. 4F); median lobe with dorsal plate abruptly narrowed from subapex and ventral plate narrowed from subapex (Fig. 5H); basal struts half length of median lobe (Fig. 5E); spermatheca (Fig. 6A) short, stout, bisinuate, also bisinuate in lateral view (Fig. 6A and B).

#### Etymology

The new species is dedicated to the collector of most type specimens, Mr. Jian-Bin Huang (Nanping, CHINA), an enthusiastic amateur entomologist. The name is a noun in the genitive case. “剑斌星天牛 (Pinyin: Jian Bin Xing Tian Niu)” is proposed for the Chinese common name of this new species.

#### Distribution

CHINA (Fujian, Guangxi).

#### Field observations

**Figure 7.**
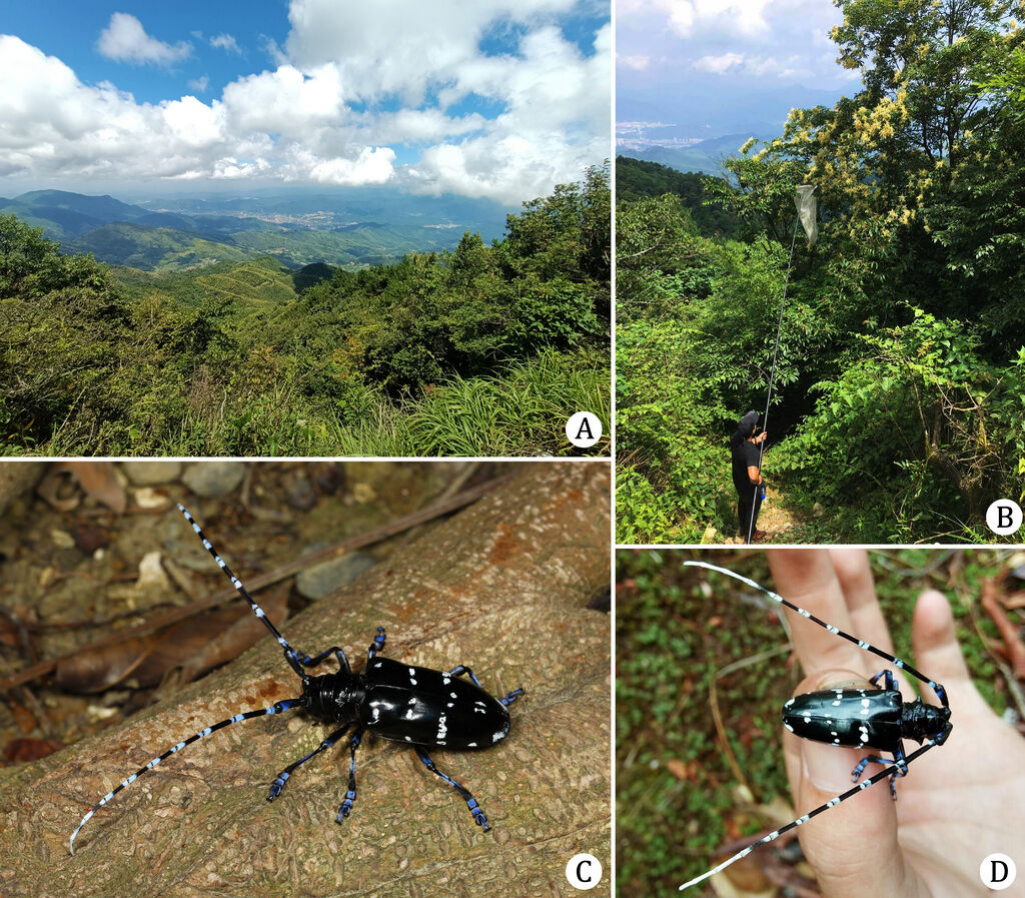
Field observations of *Anoplophorahuangjianbini* **sp. n.**: **A.** habitat of Luoboding (Fujian, CHINA); **B.** Mr. Jian-Bin Huang collecting at Luoboding (Fujian, CHINA); **C.** a living female; **D.** a living male on the palm of Jian-Bin Huang. (**A, B & D** provided by Jian-Bin Huang)

Habitat with broad-leaved mixed forest at Luoboding (Fujian) is shown in Fig. 7A and B. Living adults are shown in Fig. 7C and D.

## Supplementary Material

XML Treatment for Anoplophora huangjianbini
